# Heart Rate Variability Biofeedback in Adults with a Spinal Cord Injury: A Laboratory Framework and Case Series

**DOI:** 10.3390/jcm12247664

**Published:** 2023-12-13

**Authors:** Jacob Schoffl, Mohit Arora, Ilaria Pozzato, Candice McBain, Dianah Rodrigues, Elham Vafa, James Middleton, Glen M. Davis, Sylvia Maria Gustin, John Bourke, Annette Kifley, Andrei V. Krassioukov, Ian D. Cameron, Ashley Craig

**Affiliations:** 1 John Walsh Centre Rehabilitation Research, Northern Sydney Local Health District, Sydney, NSW 2065, Australia; mohit.arora@sydney.edu.au (M.A.); ilaria.pozzato@sydney.edu.au (I.P.); candice.mcbain@sydney.edu.au (C.M.); d.rodrigues@sydney.edu.au (D.R.); elham.vafa@sydney.edu.au (E.V.); james.middleton@sydney.edu.au (J.M.); johnny.bourke@sydney.edu.au (J.B.); annette.kifley@sydney.edu.au (A.K.); ian.cameron@sydney.edu.au (I.D.C.); a.craig@sydney.edu.au (A.C.); 2 The Kolling Institute, Faculty of Medicine and Health, The University of Sydney, Sydney, NSW 2065, Australia; 3 School of Health Sciences, Faculty of Medicine and Health, The University of Sydney, Sydney, NSW 2050, Australia; glen.davis@sydney.edu.au; 4 NeuroRecovery Research Hub, University of New South Wales, Sydney, NSW 2052, Australia; s.gustin@unsw.edu.au; 5 The Centre for Pain IMPACT, Neuroscience Research Australia, Sydney, NSW 2052, Australia; 6 ICORD, Faculty of Medicine, University of British Columbia, Vancouver, BC V6T 1Z4, Canada; andrei.krassioukov@vch.ca

**Keywords:** autonomic nervous system, psychophysiology, biofeedback, heart rate, spinal cord injuries, case reports

## Abstract

Heart rate variability biofeedback (HRV-F) is a neurocardiac self-regulation therapy that aims to regulate cardiac autonomic nervous system activity and improve cardiac balance. Despite benefits in various clinical populations, no study has reported the effects of HRV-F in adults with a spinal cord injury (SCI). This article provides an overview of a neuropsychophysiological laboratory framework and reports the impact of an HRV-F training program on two adults with chronic SCI (T1 AIS A and T3 AIS C) with different degrees of remaining cardiac autonomic function. The HRV-F intervention involved 10 weeks of face-to-face and telehealth sessions with daily HRV-F home practice. Physiological (HRV, blood pressure variability (BPV), baroreflex sensitivity (BRS)), and self-reported assessments (Fatigue Severity Scale, Generalised Anxiety Disorder Scale, Patient Health Questionnaire, Appraisal of Disability and Participation Scale, EuroQol Visual Analogue Scale) were conducted at baseline and 10 weeks. Participants also completed weekly diaries capturing mood, anxiety, pain, sleep quality, fatigue, and adverse events. Results showed some improvement in HRV, BPV, and BRS. Additionally, participants self-reported some improvements in mood, fatigue, pain, quality of life, and self-perception. A 10-week HRV-F intervention was feasible in two participants with chronic SCI, warranting further investigation into its autonomic and psychosocial effects.

## 1. Introduction

Spinal cord injury (SCI) is a chronic condition requiring substantial rehabilitation and personal adjustment [1]. In addition to the loss of motor and sensory function resulting in disability, autonomic nervous system (ANS) disruption is prevalent following an SCI [2,3]. The ANS is the part of the nervous system responsible for maintaining a homeostatic state by eliciting fine control over the body’s internal functions, including the heart, lungs, vasculature, bowel, bladder, and reproductive organs [4]. Following an SCI, neural pathways in the ANS, particularly sympathetic pathways in high-level injuries, are often disrupted, affecting the functioning of innervated organs [5]. Disruption of autonomic function is a significant contributor to severe cardiovascular complications, such as autonomic dysreflexia and postural hypotension, as well as secondary health conditions, including fatigue, psychological disorders, and cognitive impairment [2,3,5,6,7,8,9]. These conditions negatively impact on the quality of life (QoL) and social participation of those living with SCI and can contribute to a substantial financial burden [10,11]. Hence, interventions focusing on improving autonomic function can potentially improve health and QoL for individuals with SCI.

Heart rate variability biofeedback (HRV-F) is a self-regulation intervention that has shown effectiveness in improving cardiac autonomic function and physical and mental health in able-bodied populations [12]. Heart rate variability (HRV) is a cardiac autonomic marker reflecting changes in time intervals between beat-to-beat cycles of the heart, which can provide insights about overall health and a person’s ability to self-regulate [13,14]. Higher HRV is associated with better cardiovascular health, reduced stress, greater emotional regulation, and better cognitive function [13,15]. In those with an SCI, HRV is reduced and is associated with increased fatigue [9]. HRV-F aims to maximise HRV through paced breathing at an individual’s resonant frequency. Resonant frequency (e.g., 6 breaths/minute) refers to the respiration rate that synchronises the body’s natural cardiovascular oscillations (i.e., heart rate and blood pressure), generating large amplitude cardiac oscillations and maximising HRV [16,17]. Enhancing HRV through this training has yielded various benefits, such as improved blood pressure regulation, pulmonary function, cognitive function, emotional regulation, physical performance, and reduced somatic symptoms like pain and fatigue [12]. These beneficial effects have been observed in non-neurological populations, such as in individuals with asthma [18], and populations with neurological conditions, such as individuals who have experienced an acute stroke [19], fibromyalgia [20], or a traumatic brain injury [21], using similar HRV-F protocols with different time periods (2 weeks to 3 months).

One of the primary mechanisms behind HRV-F and its beneficial health effects is the increase in baroreflex sensitivity (BRS) [16]. The baroreflex is responsible for maintaining blood pressure homeostasis by modulating both the heart rate (vagal component) and total peripheral vascular resistance (sympathetic component) [22]. The term BRS identifies the effectiveness of the baroreflex in detecting and compensating for changes in blood pressure [23] and commonly refers to its vagal component (i.e., the cardiac–vagal baroreflex function) [24]. The baroreflex is stimulated during resonant frequency breathing as paced respiration induces heart rate and blood pressure oscillations that must be regulated to maintain a stable BP. Biofeedback, using signals such as respiration and heart rate/HRV, empowers individuals to control their physiology during this breathing to maximise cardiorespiratory (respiration and heart rate) and cardiovascular (heart rate and blood pressure) resonant properties [25,26]. By achieving resonance regularly, it is believed that baroreflex sensitivity can be trained and enhanced.

While HRV-F may potentially improve rehabilitation outcomes and QoL following a SCI, there is a lack of evidence concerning the effectiveness of HRV-F in adults with SCI. This study presents preliminary data from the first two participants in a pilot study of HRV-F intervention for people with SCI. To evaluate autonomic and neural function in adults with an SCI following an HRV-F intervention, The Neuropsychophysiological Laboratory for People With Spinal Cord Injury was established, and an assessment protocol was developed [27]. This paper has two primary aims: (A) to present a thorough overview of our laboratory framework and (B) to present the preliminary results of two participants who participated in the pilot phase of a future randomised controlled trial.

## 2. Materials and Methods

A laboratory framework and pilot data from two adults with chronic SCI related to investigating the effectiveness of HRV-F are presented. These participants were part of the pilot phase of a randomised controlled trial prospectively registered with the Australian and New Zealand Clinical Trial Registry (ACTRN12621000870853), with the trial protocol published elsewhere [27]. Written informed consent was provided before they participated in the study. A COVID-19 safety protocol was adhered to during the trial.

### 2.1. Laboratory Framework

**Figure 1:**
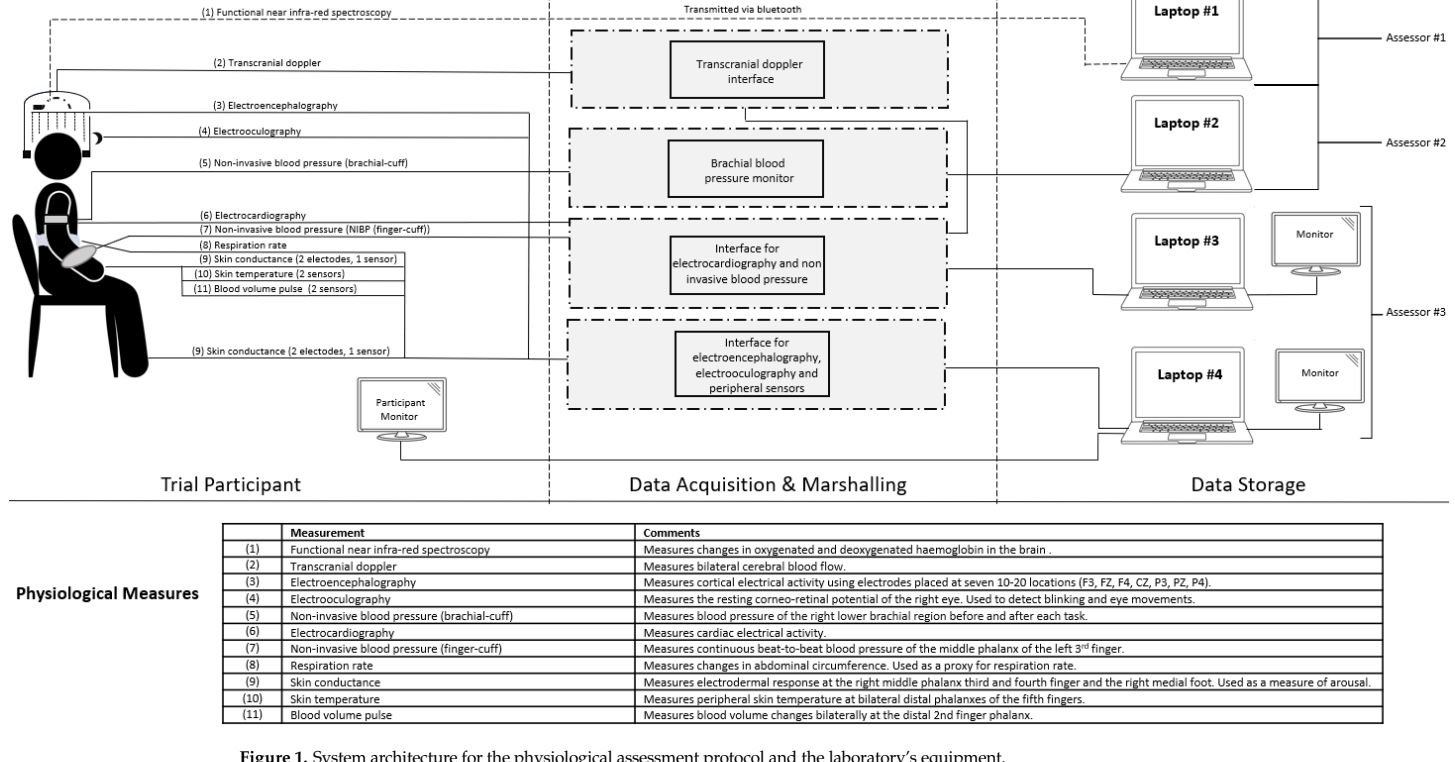
System architecture for the physiological assessment protocol and the laboratory’s equipment.

The Neuropsychophysiological Laboratory for People With Spinal Cord Injury was established in 2021. The laboratory has a range of specialised technology to comprehensively assess autonomic and neural function in adults with SCI. These include transcranial Doppler (for evaluating cerebral blood flow), electroencephalography (for assessing brain electrical activity), electrocardiography (ECG, for assessing cardiac electrical and vagal activity), non-invasive blood pressure (NIBP, for measuring blood pressure fluctuations and sympathetic adrenergic activity), electrooculography (for assessing the corneo-retinal potential), skin conductance (for assessing arousal and sympathetic sudomotor activity), body surface temperature (for monitoring thermal changes), functional near infra-red spectroscopy (for capturing changes in haemoglobin concentration), and respiration (for capturing patterns of respiration). This equipment captures physiological responses and provides valuable insights into autonomic regulation (i.e., vagal and sympathetic activity) and overall health. Figure 1 provides a detailed overview of the laboratory’s equipment architecture and how data are collected during physiological assessments.

### 2.2. Case Series

Participants were recruited from the community via an SCI Unit database, SCI consumer newsletters, newspaper advertisements, referrals from outpatient physicians, and flyers distributed in hospital and wheelchair sports facilities. Inclusion criteria were as follows: (i) 18–80 years of age; (ii) English speaking; (iii) sustained an SCI of any level from traumatic or non-traumatic cause with either complete or incomplete lesions; and (iv) at least 12 months post-injury. Neurological level of injury and AIS were determined using the International Standards for Neurological Classification of Spinal Cord Injury (ISNCSCI). The exclusion criteria were as follows: (i) evidence of severe cognitive impairment (i.e., moderate to severe traumatic brain injury or dementia); (ii) evidence of psychiatric disorder (e.g., bipolar disorder or psychoses or severe depressive disorder), as determined by medical history or psychological screening (e.g., PHQ-9); (iii) taking β-blockers.

#### 2.2.1. Study Design

This case series presents the pilot pre-post data of two adults with chronic SCI who received a 10-week HRV-F intervention.

#### 2.2.2. Assessment and Outcome Measures

Both participants were assessed at baseline (Day 0) and post-intervention (Week 10). The assessment comprises two parts: (i) self-reported and (ii) physiological assessment.

Participants completed a self-reported questionnaire via a secure web platform (REDCap (2023), V12.5.8, Vanderbilt University). This included questions about demographic and injury characteristics and psychosocial outcomes such as mood, anxiety, secondary health conditions, appraisal of injury, pain, fatigue, and QoL (see Supplementary Table S1 for more details).

The physiological assessment comprised a 5-stage physiological assessment protocol (29-min). ECG and NIBP (finger and brachial) were continuously collected during the assessment and used to calculate HRV, blood pressure variability (BPV), and baroreflex sensitivity (BRS). A minimum of five minutes of data were recorded for each stage, according to the minimum standard requirements specified in HRV guidelines [28]. The five stages are shown below:

(i) A resting condition, preceded by two minutes of habituation.
(ii) A mental stressor task (i.e., Stroop test). The Stroop test is a widely used cognitive stressor [29,30].
(iii) A recovery period.
(iv) A paced breathing task. This involved participants following a pacer, presented on a monitor display, to regulate their breathing to 6 breaths per minute. The inhalation–exhalation ratio was 1:1, with nil breath holds during this task.
(v) A second recovery period.

To minimise the effect of factors known to affect HRV, before the physiological assessment, participants were instructed to adhere to the following guidelines: regular sleep routine, no caffeine/alcohol/exercise/smoking, or large meals at least 2 h before. The assessment was conducted between 9 a.m. and 1 p.m. in a quiet, semi-darkened laboratory at a constant temperature of 23–24 degrees Celsius. Participants were also asked to empty their bladder/bowel immediately before the assessment and remained seated in their wheelchairs for the duration of the whole assessment (approximately 3 h).

#### 2.2.3. Intervention

The 10-week intervention involved six weekly face-to-face sessions conducted in the laboratory lasting between 2 and 3 h per session and four weekly telehealth sessions (via phone call) lasting between 15 and 30 min. Table 1 provides an overview of the HRV-F program. The program was focussed on HRV-F, where participants performed slow diaphragmatic breathing at their optimal resonant frequency, using biofeedback technology to synchronise their respiration and heart signals [23]. The resonant frequency assessment was based on a protocol described elsewhere [31,32]. This involved assessing several respiration rates (5–8 breaths/minute) and identifying which frequency maximised HRV measures and synchronisation between heart rate and respiration oscillations. Further details regarding the resonant frequency assessment criteria may be found in Supplementary Table S2.

**Figure 2:**
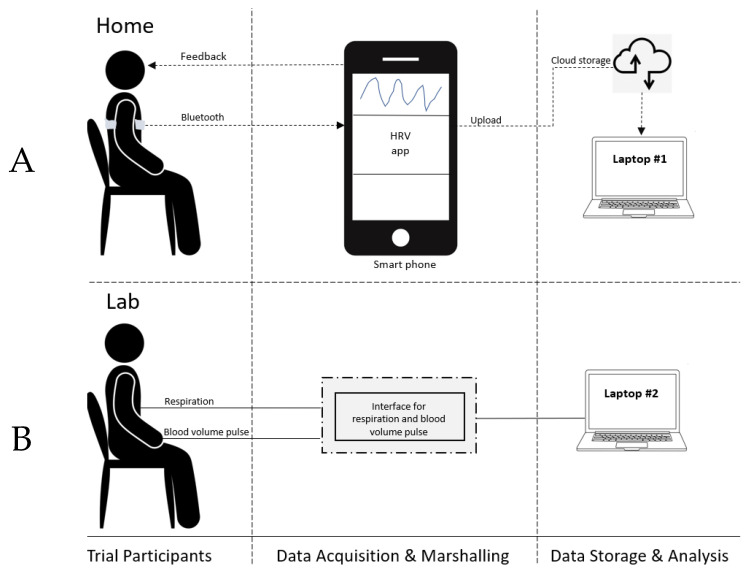
System architecture for the biofeedback intervention. (**A**): Set-up for home practice. (**B**): Set-up for laboratory. The same laboratory set-up is used for the resonant frequency assessment and the in-lab biofeedback practice. HRV: Heart rate variability.

In addition to the 10 weekly HRV-F sessions, the participants were asked to practise HRV-F at home twice daily for 20 min [33]. HRV-F home practice was conducted independently by the participants (no clinicians) using equipment provided to them during the laboratory sessions. Participants were provided with a Polar H10 Heart Rate Monitor (Polar Electro Oy, Kempele, Finland) to record heart rate/HRV signals to guide biofeedback training. This was paired with an HRV feedback application on the participant’s smartphone (Figure 2), with the respiration pacer set to the participant’s resonant frequency. The telehealth sessions were used to check in with the participant and to troubleshoot any issues the participant may have with the home practice. Telehealth sessions were separate from home practice and no HRV-F practice was performed during these telehealth sessions.

**Table 1:** An overview of the intervention program.

Week	Contents and Visit Type
1	Introduction (laboratory) Psychoeducation: What is HRV-F, physiological links between mind/body, diaphragmatic breathing Familiarisation with biofeedback and home training equipment Resonant frequency breathing (RFB) assessment
2	Mindful breathing (laboratory) Education: Using RFB in everyday contexts Mindful breathing HRV-F practice
3	Visualisation strategies (laboratory) Education: Visualisation and mental rehearsal exercises HRV-F practice
4	Debrief (phone call) Debrief and reflect upon the past three weeks Troubleshoot any homework issues
5	Mindfulness (laboratory) Mindfulness strategies and techniques RFB reassessment HRV-F practice
6–8	Debrief (phone call) Debrief and reflect upon the previous week Troubleshoot any homework issues
9	Goal setting (laboratory) Goal setting and flow state RFB reassessment HRV-F practice
10	Overview (laboratory) Overview of the program Where to from now? RFB beyond the program HRV-F practice

#### 2.2.4. Analyses

##### Self-Reported Outcomes

Self-reported questionnaires were completed via REDCap. These values were exported and tabulated. These outcomes were compared to normative data from other studies to reference whether the change in outcome was significant (see Table 2).

##### Physiological Outcomes

Kubios HRV Premium Analysis software (Version 3.5.0, Department of Applied Physics, University of Kuopio, Kuopio, Finland) was used for ECG signal preprocessing and HRV analysis in the time and frequency domain. Root mean square of successive differences (RMSSD), a time-domain measure of variability between consecutive beats, was used as a measure of cardiac vagal activity [14]. Frequency domain measures included low frequency (HRV-LF; 0.04–0.15 Hz) and high-frequency (HRV-HF; 0.15–0.4 Hz) power. HRV-LF power reflects a combination of sympathetic and parasympathetic activity upon the sinoatrial node and is suggested to reflect the cardiac-vagal baroreflex function [16]. HRV-HF power is another index of cardiac vagal activity correlated with RMSSD [14], with lower values associated with stress and anxiety [14].

Systolic blood pressure (SBP) signals were analysed using a custom-written program (MATLAB R2021b, MathWorks, Portola Valley, MA, USA) to calculate beat-to-beat BPV [34]. SBP low-frequency power (SBP-LF [0.04–0.15 Hz]) was used as a non-invasive estimate of the sympathetic control of vasculature [35].

Cardiovagal BRS was assessed using the sequence method [36] in a custom-written MATLAB program. The sequence method identifies sequences of >3 consecutive beats where a progressive shortening/lengthening of beat-to-beat intervals occurs as a function of progressive increasing/decreasing SBP. BRS gain was calculated as the average of the transfer function of these sequences. Baroreflex effectiveness index (BEI) was calculated as the number of identified sequences divided by the total number of systolic-only identified sequences (where a sequence of progressive increases/decreases in systolic BP occur without concurrent shortening/lengthening of heart beats).

**Table 2:** Psychosocial measures at baseline and 10-week assessments. Normative values from other studies are presented for reference.

	P1		P2		Normative Values		
	0 w	10 w	0 w	10 w	Population	Mean	SD
GAD-7	1	4	0	0	* SCI population (n = 465) [37]	3.86	4.34
PHQ9	8	8	0	2	* SCI population (n = 116) [38]	5.23	7.451
FSS	6	5	1.89	2.89	Chronic SCI—no cognitive impairment (n = 53) [39]	3.55	1.35
EQ-VAS	60	76	75	81	General Danish population (n = 1012) [40]	82.43	15.89
ADAPSS—SF	19	16	27	19	* SCI population (n = 256) [41]	16.32	6.84
ISCIPDS— Pain intensity	8	8	5	2	Chronic SCI—Non-neuropathic pain (n = 290) [42]	5.67	2.28
ISCIPDS— Activities	7	4	2	0	Chronic SCI—Non-neuropathic pain (n = 290) [42]	3.73	3.17
ISCIPDS— Mood	7	3	1	0	Chronic SCI—Non-neuropathic pain (n = 290) [42]	3.16	3.11
ISCIPDS— Sleep	5	6	5	1	Chronic SCI—Non-neuropathic pain (n = 290) [42]	3.64	3.41

ADAPSS-SF: Appraisal of Disability and Participation Scale- short form; EQVAS: EuroQol Visual Analog Scale; FSS: Fatigue Severity Scale; GADS: Generalised Anxiety Disorder Scale; ISCIPDS: International Spinal Cord Injury Pain Basic Data Set; P1: Participant 1; P2; Participant 2; PHQ9: Patient Health Questionnaire; 0 w: Baseline assessment; 10 w: 10-week assessment. Higher scores on GAD-7, PHQ-9, FSS, and ISCIPDS–pain intensity scales correspond to greater anxiety, depression, fatigue, and pain intensity, respectively. Lower scores on the ADAPSS-SF indicate a more positive appraisal of injury. Higher scores on the ISCIPDS-activities, ISCIPDS-mood, and ISCIPDS-sleep scales indicate a greater interference caused by pain in these aspects of life. Higher scores on the EQ-VAS indicate an improved quality of life. * SCI population is comprised of different injury levels and time since injury.

Further details regarding HRV, BPV, and BRS analyses may be found in Supplementary Table S1. HRV, BPV, and BRS analyses are presented for the resting condition, Stroop test, and paced breathing events to reflect changes in autonomic function during these tasks. Recovery data can be found in Supplementary Table S3.

## 3. Results

### 3.1. Case Presentations

Participant #1 (P1), aged 58 years, had a non-traumatic SCI (T1 AIS A), and was 3 years post-injury. P1 self-reported no previous mental health concerns, a vocational education, minimal alcohol consumption (1–2 drinks/3 months), no consumption of illicit drugs, and mobilised in a motorised wheelchair. Their medications included amitriptyline (25 mg/day), pregabalin (125 mg/morning, 75 mg/3 p.m., 75 mg/evening), and tylenol (3 × 1000 mg/day).

Participant #2 (P2), aged 54 years, had a non-traumatic SCI (T3 AIS C), and was over 25 years post-injury. P2 reported no previous mental health concerns, a vocational education, minimal alcohol consumption (1–2 drinks/3 months), no consumption of illicit drugs or any medications, and mobilised in a manual wheelchair.

### 3.2. Psychosocial Measures

Table 2 reports the psychosocial measures from the baseline and 10-week assessments. Normative data from other studies have been provided for reference. The EQ-VAS, ADAPSS-SF, and ISCIPDS indicated improvements in participants’ quality of life, appraisal of injury, and pain interference. A decrease in fatigue for P1 and increase in fatigue for P2 were evident from FSS. A slight increase in anxiety, as shown by the GAD7 was seen for P1, with no change for P1 and an increase in depression for P2 was indicated by the PHQ9; although their scores were not clinically significant, remaining below 10.

### 3.3. Physiological

#### 3.3.1. HRV, BPV, and BRS

Table 3 reports HRV, BPV, and BRS measures from the baseline and 10-week physio-logical assessments. Normative data from populations with SCI and able-bodied populations have been provided for reference. Due to the heterogeneity in acquiring and processing physiological data, comparing data from different studies should be performed with caution. P1 demonstrated little change in RMSSD during all events and an increase in HRV-HF at the 10-week assessment during the Stroop (ratio: 2.64) and paced breathing tasks (ratio: 3.07). HRV-LF only increased during the paced-breathing task (ratio: 2.10). Diastolic BP increased across all events (+4 to 9 mmHg), and systolic BP increased in the resting condition (+12 mmHg) and Stroop task (+21 mmHg) but not in the paced breathing task (−1 mmHg). SBP-LF decreased during the resting condition and Stroop task but increased during paced breathing (+1.05 mmHg^2^). BRS gain decreased slightly in the resting condition (−0.07 ms/mmHg) and Stroop task (−0.17 ms/mmHg), but increased during the paced breathing task (+0.4 ms/mmHg). BEI ratio saw improvements during the Stroop (+0.03) and paced breathing tasks (+0.13).

P2 demonstrated increases from baseline to 10-week assessment in RMSSD, HRV-LF, and HRV-HF across all physiological events, except for HRV-HF paced breathing (ratio: 0.997). Changes from baseline to 10-weeks in the the resting condition were: RMSSD (ratio: 1.94), LF power (ratio: 4.33), and HF power (ratio: 2.16). The changes in the Stroop task were: RMSSD (ratio: 2.41), LF power (ratio: 6.75), HF power (ratio: 9.24). Changes that occurred during the paced breathing task were: RMSSD (ratio: 1.67) and LF power (ratio: 6.68). Systolic BP decreased slightly during the resting condition (−2 mmHg) but increased during the Stroop (+19 mmHg) and paced breathing tasks (+10 mmHg). Diastolic BP increased during the resting condition (+7 mmHg), slightly decreased during the Stroop task (−2 mmHg), and did not change during the paced breathing task. SBP-LF power decreased for all events. BRS gain increased during resting (+0.94 ms/mmHg) and the paced breathing task (+0.1 ms/mmHg) and decreased during the Stroop task (−1.82 ms/mmHg). BEI ratio improved across all events (resting: +0.3, Stroop: +0.25, paced breathing: +0.38).

**Figure 3:**
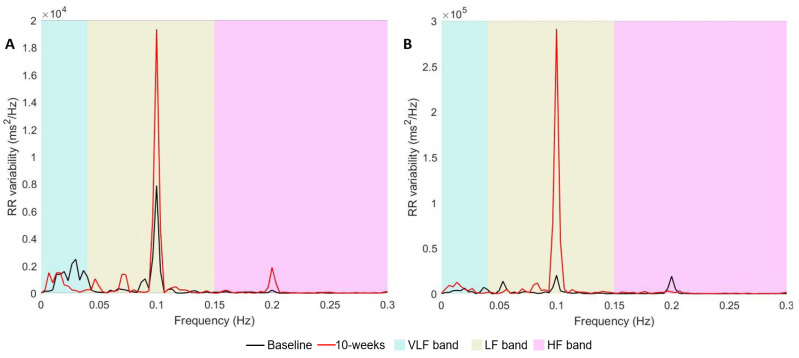
Heart rate variability power spectrum during paced breathing for Participant 1 (**A**) and Participant 2 (**B**) at baseline and 10 weeks post HRV-F. VLF band: very-low frequency band (0–0.04 Hz); LF band: low-frequency band (0.04–0.15 Hz); HF band: high-frequency band (>0.15 Hz); RR variability: variation in time period between beat-to-beat intervals of the heart.

Figure 3 displays the power spectrum (HRV-LF and HRV-HF bands) at baseline and 10 weeks for both participants during paced breathing. During this condition, a peak around 0.1 Hz within the HRV-LF band is desirable as it indicates effective cardio-respiratory resonance and enhanced baroreflex function [16]. Both P1 and P2 demonstrated significant increases in this peak.

**Table 3:** Heart rate variability, blood pressure variability and baroreflex sensitivity measures for baseline and 10-week assessment.

	Baseline			10-Weeks			Normative Values under Resting Conditions	
	HRV							
	R	S	PB	R	S	PB	Mean (SD)	Population
P1								Chronic SCI T3 and above (*n* = 21) [43]
RMSSD (ms)	7.28	3.86	4.69	5.13	4.36	6.96	29.8 (24.3)	
HRV-LF (ms^2^)	116.19	8.82	60.45	40.07	7.22	127.08	285.4 (325.5)	
HRV-HF (ms^2^)	38.61	3.97	5.46	25.6	10.50	16.74	513.4 (879.4)	
P2								
RMSSD (ms)	20.82	23.51	18.66	40.49	56.64	31.11	29.8 (24.3)	
HRV-LF (ms^2^)	349.43	318.00	254.21	1512.98	2146.35	1698.77	285.4 (325.5)	
HRV-HF (ms^2^)	186.92	201.84	131.75	403.69	1865.93	131.3	513.4 (879.4)	
	BPV							
	R	S	PB	R	S	PB	Mean (SD)	Population
P1								Chronic thoracic SCI (*n* = 12) [44]
BP (mmHg)	101/57	98/52	106/53	113/61	119/61	105/58	126.8 (7.0)/ 66.2 (4.1)	
SBP-LF (mmHg^2^)	5.02	2.54	2.73	4.88	2.43	3.77	17.32 (7.7)	
P2								
BP (mmHg)	111/69	107/71	105/73	109/76	126/69	115/73	126.8 (7.0)/ 66.2 (4.1)	
SBP-LF (mmHg^2^)	6.65	7.64	12.22	5.05	5.83	11.13	17.32 (7.7)	
	BRS							
	R	S	PB	R	S	PB	Mean (SD)	Population
P1								Chronic thoracic SCI (*n* = 12) [44]
BRS gain (ms/mmHg)	5.08	4.38	5.02	5.01	4.21	5.42	10.65 (3.2)	
BEI [ratio]	0.21	0.04	0.10	0.13	0.07	0.23	0.7 (0.1)	AB (*n* = 35) [45]
P2								Chronic thoracic SCI (*n* = 12) [44]
BRS gain (ms/mmHg)	7.15	9.00	7.93	8.09	7.18	8.03	10.65 (3.2)	
BEI [ratio]	0.32	0.45	0.39	0.62	0.70	0.77	0.7 (0.1)	AB (*n* = 35) [45]

BP: Brachial blood pressure; BEI: Baroreflex Effectiveness Index; BRS gain: Vagal Baroreflex Sensitivity; HRV: Heart rate variability; HRV-HF: HRV high-frequency power; HRV-LF: HRV low-frequency power; AB: Able-bodied population; PB: Paced breathing; P1: Participant 1; P2: Participant 2; R: Resting condition; RMSSD: Root mean square of successive differences; S: Stroop test; SBP-LF: Systolic low-frequency power.

#### 3.3.2. HRV-F Results

Both participants had an initial resonant frequency of 6 breaths/minute with an inhalation–exhalation ratio of 4 s:6 s. Over the intervention, this ratio was slightly modified in both participants (increased inhalation period) according to patient comfort and fatigue levels. Supplementary Figure S1 provides data for each individual’s baseline resonant frequency assessment.

P1 practised HRV-F at home for approximately 34 h over the 10 weeks with an adherence rate of twice daily HRV-F of 76% (102 out of 134 biofeedback sessions). P2 practised HRV-F at home for approximately 24 h with an adherence rate of 54% (72/134).

#### 3.3.3. Self-Reported Weekly Diaries

Over the 10 weeks, small trends were noted in the participants’ weekly diaries. Both participants trended towards a slight improvement in mood. P1 trended towards a slight decrease in anxiety and pain, whereas P2 trended towards a slightly larger decrease in anxiety and pain. Both participants trended towards reduced sleep quality.

Over the course of the intervention, both participants reported several life and adverse events. Life events included hospitalisation of family members, issues with pets, and housing rearrangements which were reported to affect adherence to the twice-daily HRV-F home practice. Adverse events included fatigue, difficulty sleeping, and low energy. Whilst these did not occur during HRV-F home practice, they may have been directly related to the intervention.

Despite these events, both participants reported enhanced body awareness, improved pain management, and a better ability to manage stress. P1 also reported increased confidence and independence in the community. For example, being able to take public transport independently for the first time since their injury.

## 4. Discussion

This case series demonstrated preliminary evidence for the effect of a 10-week HRV-F intervention in two adults with chronic T1 AIS A and T3 AIS C injuries, respectively. While we only present two cases, and therefore any conclusions are limited in terms of generalising to the larger population, our findings showed that HRV-F was associated with changes in cardiac autonomic regulation, appraisal of injury, and health-related QoL for these two adults with chronic non-traumatic SCI.

### 4.1. Cardiovascular Changes

Cardiac autonomic regulation improved to varying extents for each individual. Given many factors influence cardiac autonomic regulation, including seasonal variation and circadian rhythm, it is difficult to identify which factors are responsible for this. We hypothesise that the varying degrees of remaining cardiac autonomic control for each participant may have played a significant role in this. The greatest improvement was shown by P2, who had a lower-level incomplete injury and would be expected to have a greater preservation of cardiac innervation compared to P1. P2 showed increases in HRV and BEI measures across all conditions. These improvements are not unexpected, given that similar outcomes have been found in both healthy, able-bodied individuals and diverse disease populations [12,46]. Preservation of cardiac autonomic innervation should be explored further as a possible determinant of how individuals with SCI respond to HRV-F.

In contrast, P1 saw a decrease in some HRV measures during the resting condition and Stroop tasks (Resting condition: RMSSD, HRV-LF, HRV-HF. Stroop: HRV-LF), which differs from the previous literature and the hypothesised effect of HRV-F [46]. These findings need clarification with a larger sample with more diverse injury characteristics and may be confounded by concurrent medical and psychosocial factors. Factors such as level of injury, time since injury, completeness of injury, amount of exercise, and medication use differed between participants and may also contribute to the disparity between these participants. Beyond being a marker of cardiac function, HRV is negatively associated with psychosocial factors such as stress and anxiety [13]. Life events experienced by P1, such as family concerns and the hospitalisation of family members, may increase stress and decrease HRV [47].

Nevertheless, P1 demonstrated increases in most HRV and BRS measures, such as RMSSD, HRV-LF, HRV-HF, and BEI, during the paced breathing task. This task appears to provide optimal conditions for cardiorespiratory and cardiovascular resonance and maximise HRV/autonomic changes [48]. Figure 3 depicts the increase in HRV power for both participants during the paced breathing task, represented as a single peak within the HRV-LF region, at the 0.1 Hz frequency (corresponding to 6 breaths/minute). Around this frequency range, it is known that the baroreflex reaches resonance with respiration, a phenomenon referred to as ‘baroreflex resonance’ [16]. Whilst it is difficult to control for confounders in a case series design, these observed changes may likely be vagally mediated [14] and suggest the potential for HRV-F to improve cardiac autonomic regulation, especially under optimal conditions such as paced breathing [49]. However, it is important to acknowledge that various factors may also have contributed to this change, such as environmental factors (i.e., seasonal changes, circadian rhythm) and technical factors (i.e., participants learning to follow the pacer correctly).

Blood pressure changes (absolute values) between tasks trended towards a physiological response that would be expected in a healthy population. This was reflected in an increase in the value in response to a cognitive stressor (Stroop task) and a decrease in response to paced breathing. Blood pressure is typically reduced in the SCI population due to several factors, including a reduced vasomotor tone and reduced vascular muscle pump, which becomes more evident in higher-level injuries [3]. Poor blood pressure management contributes to complications such as orthostatic hypotension [3]. A larger sample is needed to validate these findings and investigate the effect that HRV-F may have on the occurrence of these complications.

SBP-LF provides a measure of vascular sympathetic activity [44]. In an able-bodied population, lower blood pressure variability is desirable as it leads to a more stable BP and reduced adverse cardiovascular events [50]. In contrast, lower SBP-LF in an SCI population has previously been shown to mirror reduced sympathetic activity and potentially more significant disruption of autonomic control [35,44]. Recently, Lucci and colleagues showed that a cutoff of <2 mmHg of SBP-LF could be used to identify adults with an autonomic complete SCI [34]. Whilst both participants in the current case series possessed autonomic incomplete injuries (cutoff > 2 mmHg^2^), they had different degrees of cardiac autonomic control. Minimal change in SBP-LF was observed for P1 whereas a reduction was noted for P2. Many factors could be responsible for these changes and it is not possible to conclude these changes were the result of HRV-F. Further investigation is needed to evaluate ideal SBP-LF values in a population with SCI and the impact that HRV-F has on blood pressure variability. It may be that for incomplete lower-level lesions, a lower SBP-LF is desirable whereas higher SBP-LF may be appropriate for complete higher-level lesions.

### 4.2. Psychosocial Changes

Previous studies have highlighted that as little as 2–4 weeks of HRV-F effectively improves psychological health in various populations [46]. In this case series, minimal change was noted in the psychological measures for anxiety and depressive mood. Whilst the *n* = 2 case series limits our ability to draw conclusions, this may be due to participants scoring low-to-mild levels of depressive mood and anxiety at baseline. It has been previously shown that biofeedback can be more effective for those with psychopathology [51]. We hypothesise that certain sub-groups of the SCI population with psychological problems would see more significant improvements in mood following HRV-F. By identifying groups that respond positively to HRV-F, personalised treatment plans and better patient outcomes can be achieved.

By contrast, improvements were shown in other psychosocial measures, including appraisal and quality of life. These findings are aligned with P1′s self-report of improved independence in the community, independently taking public transport for the first time since their injury. Improved quality of life following HRV-F has been found in other populations, including adults with obesity and chronic neck and shoulder pain [46]. In contrast, there is minimal literature exploring the effect of HRV-F on the appraisal of injury. Positive appraisal of injury is necessary for positive adjustment following an SCI and contributes to greater quality of life [1]. Given that HRV-F is known to improve emotional regulation and promote prosocial states [52], it may be that emotions surrounding an individual’s injury are better regulated, providing a more positive outlook on the injury. Additionally, given that helpful social relationships are essential for positive adjustment following injury [1], HRV-F may promote prosocial behaviours, which contribute to the positive appraisal of injury following an SCI.

Pain is often difficult to treat and poses a significant barrier to participation for many adults with an SCI [53]. Currently, it is minimally responsive to existing non-pharmacological treatments [54]. Therefore, new non-pharmacological treatments need to be explored. In this study, HRV-F was associated with a reduction in pain intensity and the impact that pain has on daily life. These were consistent with the self-reported notes, whereby both participants reported an improved awareness of pain.

*“…say I’ve got a headache, or I am my headache, it’s kind of like I am my pain. It characterises who I am and how much I’m able to do in a day and being able to separate it as something separate to be managed has allowed me to be more productive during the day and to kind of ground myself and do more, be more active, be more independent.”*

Comment received from P1.

In addition, greater cardiac vagal activity, as measured by HRV-RMSSD and HRV-HF power, has been linked with reduced pain [55]. Similarly, HRV-F has been shown to reduce measures of pain in chronic disease populations, such as fibromyalgia and chronic shoulder and neck pain [46]. Pain may be associated with changes in the ANS, and stable improvements in pain may occur by improving cardiac vagal control via HRV-F. The reduction in sleep quality reported by both participants during the 10 weeks was unexpected and will hopefully be clarified by additional follow-up and a larger sample size.

### 4.3. Considerations for Future Research

For future investigations exploring HRV-F, dosage is a key consideration. Participants were encouraged in the current case series to perform 20 min of HRV-F home practice twice daily, which was often difficult for them to achieve. Nevertheless, some benefits were still achieved by both participants. In a review of studies with chronic disease populations, Fournie and colleagues found improvements from HRV-F in as little as 4 weeks, and with home practice for as little as 15 min a day [46]. Our findings indicate that some reorganisation in the cardiac ANS can occur in as little as 10 weeks, however, it may only be apparent under controlled physiological conditions (e.g., slow/paced breathing). Whilst P1 completed more sessions of HRV-F home practice than P2, greater cardiac autonomic benefits were noted by P2. Therefore, the optimal dosage of HRV-F for certain benefits needs clarification, as does the impact of the injury aetiology, level of injury, degree of remaining cardiac autonomic control, psychosocial status, and time since injury.

Another consideration is the role of inhalation–exhalation ratios. It is established that a longer exhalation increases vagal efferent activity, resulting in a greater parasympathetic response [56]. Previous studies have found that a lower ratio is associated with greater relaxation and less stress [57]. In able-bodied populations, dominant sympathetic activity tends to be an important consideration. This is generally opposite to the state found in a SCI population with high-level lesions. Therefore, customisation of this ratio for each individual may be essential to realise further benefits, and future studies should investigate how these ratios affect HRV-F outcomes.

### 4.4. Limitations

There are several limitations to this study. Firstly, this case series included two participants only, limiting our ability to draw conclusions and generalise findings to the broader SCI population. The current study is not intended to be robust enough to reflect a proof-of-concept for the effectiveness of HRV-F. Rather, it is essentially a pilot phase, which highlights that HRV-F is a feasible intervention and has the potential to provide therapeutic and possibly diverse benefits to two participants with different SCI characteristics. This suggests that it is worth pursuing in a larger randomised controlled trial. Given the heterogeneity of a population with SCI, a larger sample size with a greater representation of SCI characteristics, such as time since injury, level of injury, aetiology of injury, and completeness of injury, is warranted for more generalisable results. Another area for improvement is the need for standardisation of home biofeedback practice. Whilst participants were instructed to perform HRV-F in a seated position with eyes open, various other factors such as circadian rhythm, meals/caffeine consumption, and amount of exercise and sleep could influence HRV results. However, placing additional restrictions would arguably impact adherence to HRV-F practice. Furthermore, using the Elite HRV app, we were unable to verify if participants followed the visual respiration pacing correctly during their home practice. Lalanza and colleagues recommended using respirometers for this issue; however, these can be costly [58]. Furthermore, respiration rate is known to influence HRV in a SCI population [59], which may confound HRV results. Whilst we did not restrict respiration rate during the assessment, a paced breathing task was added to the assessment protocol to control this.

## 5. Conclusions

A 10-week HRV-F intervention was shown to be feasible in two participants with chronic non-traumatic SCI with different injury characteristics. Varying degrees of change were found between participants, with notable changes in cardiac autonomic regulation, health-related quality of life, and perception of injury. Further research using a larger sample size with greater heterogeneity in SCI characteristics and a more extended follow-up period is required to understand the effectiveness of this intervention on adults with chronic SCI. These results provide initial support for a 10-week HRV-F intervention to be further investigated as to its autonomic and psychosocial effects on adults with chronic SCI.

## Data Availability

The data presented in this study are available on request from the corresponding author. The data are not publicly available to ensure participant privacy.

## Supplementary Materials

The following supporting information can be downloaded at: https://www.mdpi.com/article/10.3390/jcm12247664/s1, Table S1: Description of presented measures; Table S2: Criteria for the resonant frequency assessment; Table S3: HRV, BPV and BRS outcomes for all physiological events; Figure S1: Baseline resonant frequency assessments for P1 and P2. References [60,61,62,63,64,65,66] are cited in the Supplementary Materials.
