# Supplementary material for: Heart Rate Variability Biofeedback in Adults with a Spinal Cord Injury: A Laboratory Framework and Case Series
Source: J Clin Med. 2023 Dec 13;12(24):7664. doi: 10.3390/jcm12247664 (PMC10743967; doi:10.3390/jcm12247664)
Supplement: Supplementary file 1 [file jcm-12-07664-s001.zip › jcm-2594846-supplementary - updated.pdf]

Table S1. Description of presented measures. Among the battery of measures collected during the larger trial [27], only the following psychosocial and physiological outcomes are presented for this case series. Measurements were collected at baseline (pre-intervention) and 10-weeks (post-intervention). All measures used have demonstrated reliability and validity [27].

Outcomes measures	Description
Generalised Anxiety Disorder Scale (GAD-7)	The GAD-7 is a validated screening tool for assessing the severity of anxiety and associated symptoms (restlessness, fatigue, rumination) over the previous 2 weeks [60]. The scale has seven items, scored from 0-3, with a total score range of 21. Higher scores indicate higher anxiety levels.
Patient Health Questionnaire (PHQ-9)	The PHQ-9 is a validated tool for assessing the severity of depressive symptoms [61]. The questionnaire covers depressive mood symptoms such as loss of interest in activities, feelings of sadness, and thoughts of self-harm. The questionnaire has nine items, scored from 0-3, with a total score of 27. Higher scores indicate higher levels of depressive symptoms
Fatigue Severity Scale (FSS)	The FSS is a valid tool designed to assess fatigue and its impact on daily activities [62]. It has nine items, scored from 1-7, which are summed together and divided by nine. Higher scores indicate greater levels of fatigue.
EuroQol Visual Analogue Scale (EQ-VAS)	The EQ-VAS is a self-report tool with sufficient construct validity for assessing quality of life [63]. The EQ-VAS consists of a 100-point Likert scale with "0" indicating "worst health" and "100" indicating "best health". Participants select a point on the scale that they feel correlates to how they would rate their health on that particular day
Appraisal of Disability and Participation Scale – Short Form (ADAPSS-SF)	The ADAPSS-SF is a six-item self-report tool to assess how adults with a SCI evaluate their injury [40]. Each item involves an injury appraisal statement (i.e., "Since my injury life is more frightening for me") where participants rate the degree to which they agree with the statement on a 6-point Likert scale. Higher scores indicate less adaptive appraisal mechanisms.
World Health Organisation Disability Assessment Schedule 2.0 – Participation Items (WHODAS 2.0)	The WHODAS 2.0 is a valid self-report tool to assess the extent to which individuals can participate in community life [64]. Only the participation domain is assessed. Each item is rated on a 5-point Likert scale, with scores ranging from 1 (no difficulty) to 5 (extreme difficulty), where higher scores indicate greater difficulty with participation.
Moorong Self-Efficacy Scale (MSES)	The MSES is a validated self-report tool that captures an individual's level of self-efficacy towards their health condition [65]. In other words, how confident an individual is in their ability to manage different areas of their health condition. The tool consists of 16-items, using a 7-point Likert scale, with a total score range of 18-180. Higher scores indicate greater levels of perceived self-efficacy.
International Spinal Cord Injury Pain Basic Data Set - Interference Questions (ISCIPBDS)	The ISCIPBDS is specifically designed to capture how pain affects daily life for adults with a SCI [66]. These questions include four 10-point Likert Scales: one that captures a pain rating for the last week, and three that capture how pain interferes with daily activities, mood, and sleep (i.e., In general, how much has pain interfered with your day-to-day activities in the last week?).
Electrocardiogram (ECG)	ECG signals were measured using a modified lead II chest electrode configuration. ECG was sampled at 2000Hz, recorded using the PowerLab 35 (ADInstruments, Bella Vista, New South Wales, Australia), and acquired in LabChart v8 (ADInstruments). A band-pass filter between 0.1-150Hz was applied in LabChart to limit baseline wander and unwanted higher frequencies.
Heart rate variability (HRV)	ECG signals were exported from LabChart at a sampling rate of 2000Hz to Kubios HRV Premium Analysis software (Version 3.5.0) where HRV analysis was performed. Each ECG signal was visually inspected for noise or artefacts. Automatic beat correction and/or manual correction were used to correct ectopic beats or any misidentified ECG peaks, where appropriate. A notch filter at 50 Hz was applied to

	limit power-line noise interference. RR time series underwent cubic spline interpolation and was resampled at 4 Hz. A 3rd order polynomial filter was used to minimise the impact of slow trends (i.e., removing very low frequency components) in the ECG data. Power spectral analysis was performed using Welch's periodogram method with a window width of 300 seconds and a window overlap of 50%.
Blood pressure (BP)	Continuous non-invasive arterial BP (NIBP) signals were measured using a finger NIBP unit (ADInstruments, Bella Vista, New South Wales, Australia). A finger cuff of appropriate size was positioned around the middle phalanx of the left middle finger. NIBP was sampled at 200 Hz, recorded using the PowerLab 35, and acquired on LabChart v8 (ADInstruments, Bella Vista, New South Wales, Australia). Prior to recording and half-way through the protocol (i.e., prior to the first recovery period), NIBP autocalibration was turned on in LabChart v8 to allow the signal to achieve stability. Autocalibration was turned off during each condition to avoid missing data. Poor perfusion and temperature regulation is a common complication for adults with a SCI and can interfere with the accuracy of physiological measures [43]. To account for this, the participant's fingers were regularly checked for perfusion and heating was applied (via heat pack) to the fingers as needed. Reference brachial BP measurements (DINAMAP™ SuperSTAT, B105, GE HealthCare) were captured for calibration of the NIBP unit. Brachial BP was measured four times before recording (twice on each arm) and then immediately pre and post each condition on the right arm only (contralateral side to the NIBP unit). A systolic and diastolic BP value was reported as the BP measurement directly after the event.
Blood pressure variability (BPV)	SBP was exported from LabChart at a sampling rate of 2000 Hz to a text file. BPV analyses were performed using a custom-written MATLAB program, per the methods section's details. BP data were initially filtered using a 1st order 30Hz low pass Butterworth filter to reduce high-frequency noise. The SBP signal was aligned with the RR time series so that a corresponding SBP value was extracted for each ECG R peak. Cubic spline interpolation was performed, followed by Welch's periodogram using a Hamming window with 8 segments and a 50% overlap. The data was then resampled at 4Hz, and frequency domain calculations were performed. Power spectral analysis was performed using Welch's periodogram with a window width of 300 seconds and a window overlap of 50%.
Baroreflex sensitivity (BRS)	BRS analyses were performed using the sequence method via a custom-written MATLAB program. The sequence method quantifies changes in RR-interval as a function of SBP changes and reflects the ability of the cardiac vagal nerve to alter its influence on the SA node. BRS sequences were identified using the following criteria: >1mmHg change in successive systolic BP, >5 millisecond change in successive RR-intervals, >0.8 correlation coefficient between systolic BP and RR interval, >3 consecutive heartbeats in each sequence, and a one beat lag (delay) between systolic BP and RR-interval time series. A regression line for each sequence is then calculated and BRS was calculated as the average coefficient of these regression lines.
Elite HRV practice data	Daily HRV-F practice data were collected using the Elite HRV smartphone app paired with a Polar H10 heart rate monitor. Once a participant completed a home-based HRV-F session, the session data were automatically uploaded to the Elite HRV online team dashboard for further analysis. Elite HRV utilises its own proprietary algorithm for pre-processing and peak detection. It calculates HRV in the time domain (Standard deviation of normal-to-normal intervals and RMSSD) and performs Welch's periodogram to calculate HRV in the frequency domain (LF and HF power).
Self-Reported Weekly Diaries	During the 10-week intervention period, participants completed weekly diaries that captured psychological states, physical symptoms, and any adverse events. Mood, anxiety, pain, fatigue, and sleep quality were assessed separately using a 10-point Likert scale, reflecting the average level of each state/symptom over the past week. Participants were also asked to record any adverse events, such as autonomic dysreflexia and

	postural hypotension, that occurred during the week and how these were managed. These weekly diaries were captured on a secure online platform (REDCap).
Clinician's report	Following each training session, both clinicians discussed the participant's progress and recorded any relevant notes that the participant stated. These included any life events that may have affected practice or any progress that would not be captured by the other outcome measures. These notes were captured on a secure online platform (REDCap).

Table S2. Criteria for the resonant frequency assessment.

Criteria	Scoring
Comfort rating (/10)	Scored out of 10, with 0 indicating extreme discomfort and 10 indicating highly comfortable when breathing at the respiration rate
Max-min HR (beats/min)	A highest value is favourable. The max-min HR statistic from BioGraph Infiniti is used.
Respiration-HR phase (degrees)	A value closest to 0 is favourable, indicating that the respiration and HR are in phase (coherent). The respiration-HR phase statistic from BioGraph Infiniti is used.
Mean LF percentage of total power (%)	A higher value is favourable indicating that a greater percentage of power is located in the LF domain. The LF percentage of power statistic from BioGraph Infiniti is used.
Mean LF amplitude (milliseconds^2)	A higher value is favourable indicating greater power. The mean LF amplitude statistics from BioGraph Infiniti is used.
Mean and fewest LF peaks in spectral display (Hz)	A value around 0.1Hz is favourable. The mean LF peak statistics from BioGraph Infiniti is used. Fewer peaks are favourable and is based on visual observation of the LF spectral display.
Visual smoothness of HR curve (yes/no)	Smoother HR curves are favourable. This value is based on a visual observation of the data.

The above criteria are used to determine an individual’s resonant frequency of breathing. The respiration rate that satisfies the most criteria is usually selected. The weightings for each of these criteria are not currently validated [33] and different weightings are given to each criterion based on the clinician’s experience. In this paper, the clinician placed greater emphasis on comfort rating and respiration-heart rate phase. Whilst these criteria are the main objective measures used, the clinician also observes how the participant performs at different respiration rates, noting the style of respiration used (i.e., chest or abdominal/diaphragmatic) and the degree of labour.

Table S3. HRV, BPV and BRS outcomes for all physiological events

	Baseline					10-Weeks				
	HRV									
	**R1**	**S**	**R2**	**PB**	**R3**	**R1**	**S**	**R2**	**PB**	**R3**
**P1**										
RMSSD (ms)	7.28	3.86	5.85	4.69	5.20	5.13	4.36	4.52	6.96	5.14
HRV-LF (ms^2)	116.19	8.82	44.64	60.45	59.46	40.07	7.22	32.82	127.08	109.92
HRV-HF (ms^2)	38.61	3.97	27.33	5.46	23.54	25.6	10.50	19.10	16.74	16.34
**P2**										
RMSSD (ms)	20.82	23.51	19.25	18.66	23.56	40.49	56.64	37.18	31.11	33.9
HRV-LF (ms^2)	349.43	318.00	375.84	254.21	735.73	1512.98	2146.35	919.24	1698.77	1493.78
HRV-HF (ms^2)	186.92	201.84	116.31	131.75	157.42	403.69	1865.93	345.20	131.3	265.45
	BPV									
	**R1**	**S**	**R2**	**PB**	**R3**	**R1**	**S**	**R2**	**PB**	**R3**
**P1**										
BP (mmHg)	101/57	98/52	102/51	106/53	100/57	113/61	119/61	109/61	105/58	112/57
SBP-LF (mmHg^2)	5.02	2.54	3.31	2.73	6.71	4.88	2.43	7.34	3.78	12.03
**P2**										
BP (mmHg)	111/69	107/71	108/71	105/73	106/75	109/76	126/69	125/77	115/73	113/75
SBP-LF (mmHg^2)	6.65	7.64	5.27	12.22	3.92	5.05	5.83	2.94	11.13	2.73
	BRS									
	**R1**	**S**	**R2**	**PB**	**R3**	**R1**	**S**	**R2**	**PB**	**R3**
**P1**										
BRS gain (ms/mmHg)	5.08	4.38	4.94	5.02	4.71	5.01	4.21	5.06	5.42	5.11
BEI (ratio)	0.21	0.04	0.22	0.10	0.16	0.13	0.07	0.12	0.23	0.17
**P2**										
BRS gain (ms/mmHg)	7.15	9.00	6.76	7.93	9.3	8.09	7.18	8.49	8.03	8.73
BEI (ratio)	0.32	0.45	0.38	0.39	0.33	0.62	0.70	0.71	0.77	0.7

BP: Brachial blood pressure; BEI: Baroreflex Effectiveness Index; BPV: Blood pressure variability; BRS: Baroreflex sensitivity; HRV: Heart rate variability; HRV-HF: HRV high frequency power; HRV-LF: HRV low frequency power; PB: Paced breathing; P1: Participant 1; P2: Participant 2; R1:

Resting condition; R2: Recovery condition 1; R3: Recovery condition 2; RMSSD: Root mean square of successive differences; S: Stroop test; SBP-LF: Systolic low-frequency power.

Figure S1. Baseline resonant frequency assessments for P1 (A) and P2 (B). Comfort rating, heart rate variability, and respiration are presented for each respiration rate during the resonant frequency assessment. Yellow highlighted boxes identify ideal values for the criteria. The suggested resonant frequency is the respiration rate that satisfies the greatest number of criteria. The chosen resonant frequency is the respiration rate that the clinician decides to use, based off their own weighting of the resonant frequency criteria and visual inspection of the raw signals (respiration and blood volume pulse).

A

	Resonant Frequency Rate of Breathing: Comparison Chart							
Participant ID: P1	8	7.5	7	6.5	6	5.5	5	
	0-2mins	2-4m	4-6m	6-8m	8-10m	10-12m	12-14m	Preferred BR
Comfort rating					X			6
Max-min (beats/min)	2.9	2.9	3.6	4.1	4.3	4.6	4.8	5
Resp-HR Phase	-52	16	36	-16	4.4	29	-46	6
Mean LF % of power	76	76	76	75	71	73	70	8, 7.5, 7
Mean LF power	16.5	23.3	33.3	45.9	51.18	69.3	68	5.5
Mean and fewest LF peaks in spectral display (decimal) - LF power					X			6
Visual smoothness of HR curve (Y/N)							X	5
**Suggested resonant frequency:**								**6**
**Chosen resonant frequency :**								**6**

B

	Resonant Frequency Rate of Breathing: Comparison Chart							
Participant ID: P2	8	7.5	7	6.5	6	5.5	5	
	0-2mins	2-4m	4-6m	6-8m	8-10m	10-12m	12-14m	Preferred BR
Comfort rating			X	X				7, 6.5
Max-min (beats/min)	19.14	19.88	18.31	17.77	17.02	16.55	15.87	7.5
Resp-HR Phase	17.9	-100.5	-157.8	9	5.5	-125.8	-102.9	8, 6.5, 6
Mean LF % of power	40.16	45.52	48.13	44.08	45.7	47.47	49.32	5
Mean LF amplitude	453.05	735.79	631.79	500.4	461.04	426.67	396.05	7.5
Mean and fewest LF peaks in spectral display (decimal) - LF power	0.14	0.14	0.13	0.14	0.13	0.12	0.12	5.5, 5
Visual smoothness of HR curve (Y/N)		X			X		X	7.5, 6, 5
							**Suggested resonant frequency:**	**7.5**
							**Chosen resonant frequency :**	**6**

BR: breath rate; HR: heart rate,; LF: low frequency; N: no; Y: yes.
